# Circumferential Inferior Vena Cavectomy Without Caval Replacement in the Management of Renal Cell Carcinoma with Tumor Thrombus

**DOI:** 10.1007/s11934-024-01203-x

**Published:** 2024-05-20

**Authors:** V. Gonzalez de Gor Herrera, J. M. Asencio Pascual, J. González, F. Herranz Amo, E. LLedó García, A Sánchez Ochoa M., C. Hernández Fernández

**Affiliations:** 1 Servicio de Urología, Hospital General Universitario Gregorio Marañón, Madrid, Spain; 2 Departamento de Cirugía, Facultad de Medicina, Universidad Complutense, C/ Dr Esquerdo, 46, 28007 Madrid, Spain; 3 Servicio de Cirugía General y del Aparato Digestivo (Sección de Cirugía HBP), Hospital General Universitario Gregorio Marañón, Madrid, Spain; 4 Hospital General Universitario Gregorio Marañón, Madrid, Spain

**Keywords:** Renal cell carcinoma, Tumor thrombus, Surgery, Inferior vena cava, Circumferential cavectomy, Caval replacement

## Abstract

**Purpose of Review:**

Renal cell carcinoma presents a unique proclivity for vascular involvement giving rise to a peculiar form of locally advanced disease so-called tumor thrombus. To date, the only curative strategy for these cases remains surgery, which should aim to remove every vestige of macroscopic disease. Most of the preexisting literature advocates opening the vena cava to allow tumor thrombus removal and subsequent venous suture closure. However, inferior vena cava circumferential resection (cavectomy) without caval replacement is possible in the majority of cases since progressive occlusion facilitates the development of a collateral venous network aimed at maintaining cardiac preload.

**Recent Findings:**

Radical nephrectomy with tumor thrombectomy remains a surgical challenge not exempt of operative complications even in experienced hands. In opposition to what traditional cavotomy and thrombus withdrawal can offer, circumferential cavectomy without caval replacement would provide comparable or even better oncologic control, decrease the likelihood of operative bleeding, and prevent the development of perioperative pulmonary embolism.

**Summary:**

This review focuses on the rationale of circumferential IVC resection without caval replacement and the important technical aspects of this approach in cases of renal cell carcinoma with vascular involvement. We also include an initial report on the surgical outcomes of a contemporary series of patients managed under this approach at our center.

## Introduction

Renal cell carcinoma (RCC) accounts for 2–3% of all malignancies in adults, being the third most frequent and lethal genitourinary cancer [1, 2]. Currently, most cases of RCC are detected incidentally. However, up to 30% of them are diagnosed in locally advanced or metastatic disease stages due mainly to their long initial asymptomatic period [3]. Moreover, this entity presents a unique proclivity for vascular involvement, extending into the neighboring veins and giving rise to a special form of locally advanced involvement, so-called tumor thrombus (TT), which is present in up to 4–10% (reaching even the right atrium in 1%) of the cases[4].

TT often occupies the IVC leading to its progressive occlusion. Although the classic occluding syndrome includes the appearance of lower-limb edema, dilation of the peripheral abdominal veins, and presence of varicose deformity in the pampiniform and hemorrhoidal plexuses, the clinical presentation in real-world practice is rather variable and depends, among other factors, on the presence of patient comorbid conditions, level and degree of caval obstruction, and adequacy in the formation of a collateral venous network aimed at maintaining cardiac preload [5].

Despite the unprecedented advances in systemic treatment in the last decade, the only curative strategy for locally advanced RCC remains surgery, with overall survival rates reaching up to 68% at 5 years in the best surgical candidates [6]. Furthermore, in those patients exhibiting metastatic spreading, this option can provide a significant reduction in disease burden (which in turn can potentially improve the response to systemic treatment in a non-negligible percentage of patients) or, in the worst scenario, may provide for symptomatic relief and improvement in quality of life. Under these circumstances, surgical treatment should pursue eliminating every vestige of visible disease, including radical nephrectomy of the diseased renal unit along with the lymph nodes macroscopically involved (i.e., according to the precepts established by Robson in 1969) in conjunction with tumor thrombectomy, of both, the main thrombus located inside the IVC, and those potential dislodged fragments migrated from the latter to the pulmonary circulation.

Although surgical treatment provides encouraging results, the morbidity and mortality associated with this type of intervention remain significant, with complication rates exceeding 50% and mortality rates reaching 23.5% and 40% for infra- and supradiaphragmatic tumor thrombi, respectively [7–9]. In fact, morbidity and mortality rates are mainly impacted by two major entities: (i) perioperative bleeding and (ii) intra- and/or postoperative pulmonary embolism (PE). In addition, both surgical complications are somehow linked to the unique characteristics of the disease (i.e., high tumor volume, presence of large collateral vessels developed in response to IVC obstruction, and presence of intravascular thrombosis—of either bland or tumor thrombi) for a given patient.

Although most of the preexisting literature advocates opening the IVC (i.e., cavotomy) to facilitate tumor thrombus removal and subsequent venous suture closure, occasionally, when the development of the collateral venous network in response to occlusion is adequate and sufficient and the degree of IVC occlusion is complete/almost complete, extreme difficulty in separating the TT from the IVC wall or direct invasion is presumed, and/or a large burden of potentially uncontrollable associated bland thrombus exists, a management option to strongly consider may be to resect circumferentially the whole length of IVC affected by TT without reconstructing the venous upstream circulation towards the heart. Eventually, circumferential cavectomy avoiding caval replacement would provide comparable or even better oncologic control (by preventing local spread of disease particularly in the presence of friable TT), decrease the likelihood of bleeding (especially that associated with the opening of the IVC or its reconstruction), and prevent the development of perioperative PE (specially that related to the surgical handling of the TT and the remaining bland thrombus, since in the end, it would function as a filter placed in the infrarenal IVC).

This review focuses on the rationale of circumferential IVC resection without caval replacement and the important technical aspects of this approach in cases of RCC with vascular involvement. We also include an initial report on the surgical outcomes of a contemporary series of patients managed under this approach at our center.

## The Inferior Vena Cava System

The IVC network is the main blood-collecting venous system of the human body. In the adult, it derives from different pairs of multiple longitudinal embryonic veins through a complex process of growth, regression, and interconnections that takes place between the 6 and 8th weeks of gestation [5].

Both common iliac, phrenic, and renal veins and the right adrenal and gonadal veins flow directly into the IVC. The azygos/hemiazygos venous system is located on each side of the spine and drains the venous circulation of the visceral contents found in the mediastinum, as well as those of the dorsum and thoracoabdominal walls. This system consists of the azygos vein and its two main tributaries: the hemiazygos vein and the accessory hemiazygos vein. The azygos vein usually originates from branches emerging from the posterior aspect of the IVC or the left renal vein. Then, this vein crosses through the diaphragm, reaches the mediastinum upwards in the thorax, and finally connects with the superior vena cava. The azygos venous system forms many anastomoses with the IVC and the vertebral venous plexuses, thus making it a bridge that connects the superior and inferior vena cava and provides an alternative drainage pathway in case of IVC occlusion [10].

Renal venous drainage deserves a special consideration given the important anatomical differences established between the right and left sides. While the left kidney venous drainage is commonly shared by the main renal vein in conjunction with the adrenal vein, the gonadal vein, and rather frequently an additional posterior lumbar vein (that connects with the azygos/hemiazygos system), the right renal vein usually does not receive tributaries. Conversely, the right adrenal, gonadal, and retroperitoneal (lumbar, ascending lumbar, and hemiadrenal veins) veins flow into the right renal vein in only 30%, 7%, and 3% of the cases, respectively. Therefore, typically the left renal vein can be divided and disconnected from the IVC without major consequences. However, the right renal vein cannot be safely divided from its caval attachment unless right renal venous drainage is guaranteed by the presence of tributary lumbar veins connecting to the azygos system. Otherwise, the venous drainage of the right kidney has to be reconstructed due to the unacceptable risk of postoperative thrombosis [5].

## IVC Response to Occlusion: The Collateral Venous Network Development

RCC shows particular tropism for endovascular growth giving rise to the so-called TT. The intravascular extension of the disease is made possible only by an adequate vascular supply via at least three different mechanisms: (i) the main nutritional artery, (ii) multiple arterial neovessels—of different configurations and at different degrees of maturation—that are located within the tumor and appear in response to intratumoral proangiogenic stimuli, and (iii) directly from the ascending blood circulation through the IVC.

With progressive TT growth, the lumen of the IVC is gradually obliterated. Commonly, complete and almost complete caval occlusion carries dilation of the IVC lumen to a point, in order to give some space to the TT. Dilation of the IVC allows anterograde flow upwards to the heart until full obliteration is completed. However, the ascending flow in cases of partial occlusion is not laminar, but turbulent and may facilitate the development of bland thrombi (blood clots) that generally adhere to the TT in areas of greatest turbulence. Bland thrombus formation is also facilitated by venous ectasia and the typical hypercoagulable status of the oncologic patient.

Chronic flow obstruction facilitates the development of a network of venous collaterals to ensure the maintenance of cardiac preload to the right atrium. Moreover, the absence of clear symptoms of obstruction (IVC syndrome) usually means that the collateral network is already established. In this way, the normal upward flow through the IVC collecting blood from the lower body returns to the right heart through the different collateral systems, which basically include four main groups: deep, intermediate, superficial, and portal.

The deep azygos-hemiazygos system is the earliest channel available and plays a dominant role in venous decompression of IVC obstruction at any level. The vertebral venous plexus communicates bidirectionally with the IVC and the azygos-hemiazygos system through segmental tributaries.

In the intermediate route, blood flows through the bilateral periureteral plexus (if the veins are patent) and through the left gonadal to the left renal vein. The right flow does not usually drain as easily through the right gonadal, as it normally drains into the IVC and is usually affected by the thrombus.

Through the superficial system, abdominal blood drains from the external iliac veins to the inferior epigastric veins, creating anastomoses through the superior epigastric veins and internal mammary veins, ending in the subclavian veins. The flow is also created between the circumflex iliac veins and the superficial epigastric veins, anastomosing with the lateral thoracic and axillary veins.

Finally, in the portal system, blood from the lower limbs ascends retrogradely to the internal iliac veins through the hemorrhoidal plexus, which ascends through the inferior mesenteric vein to the splenic vein and the portal system.

The determinants of successful collateral development are the location or level of the obstructed venous segment, the length of the obstruction, and the number of veins involved. These three elements individually and in combination cause an increase in venous resistance, determining the development of collateral drainage [11].

## Diagnostic Work-up and Vascular Disease Staging

Imaging remains “la pièce de la résistance” in the diagnosis of RCC with vascular involvement, since it is not only a clear depiction of the extent of the disease, but it is also crucial during surgical planning. A recent meta-analysis in this regard indicates that both computed tomography (CT) and magnetic resonance imaging (MRI) can be considered the gold standard tests to determine the extension of RCC within the IVC. Both tests provide excellent quality images, allowing an adequate description of the relationships existing between the important anatomical and surgical landmarks and the proximal end of the tumor thrombus. Likewise, it allows for identifying the presence of potential invasion of the IVC wall, bland thrombus, and alternative venous drainage pathways [12•].

The level reached by the proximal end of the TT on preoperative imaging has historically been used to classify RCC with TT. The most widely used system remains the Mayo Clinic (MC) Classification System described by Neves and Zincke in 1987 [13]. This Classification System includes a total of four subcategories: (i) level I, the TT reaches the IVC, but extends a distance of less than 2 cm from the ostium of the main renal vein; (ii) level II, the TT extends into the IVC beyond 2 cm from the ostium of the renal vein but does not exceed the lower hepatic border; (iii) level III, TT involves the retrohepatic and suprahepatic IVC to a level below the limit provided by the diaphragm; and (iv) level IV, the TT extends to a supradiaphragmatic level, reaching the right cardiac chambers.

The MC Classification System not only provides prognostic information (i.e., since more proximal levels of TT are usually linked to larger tumors and potentially worse prognosis), but it also aids in the decision-making process regarding the surgical approach to be used, since each level requires a unique sequence of surgical maneuvers that should be better anticipated before the intervention. Accordingly, an even more precise approximation regarding the surgical maneuvers required for a given case is provided by the University of Miami (UM) Classification System described more recently by Ciancio et al. [14••]. The UM Classification System subdivides the level III category into four additional subcategories: (i) level IIIa, the TT extends to the lower edge of the confluence between the IVC and the major hepatic veins (MHVs); (ii) level IIIb, the TT reaches the confluence with the MHVs but does not exceed them; (iii) level IIIc, the TT exceeds the confluence with the MHVs, extending into the suprahepatic IVC but without exceeding the location of the diaphragm; and (iv) level IIId, the TT extends proximally towards the thorax, crossing the diaphragm and lodging inside the intrapericardial IVC, but without reaching the right atrium (i.e., level IV).

## Surgical Maneuvers According to the TT Level and Circumferential Cavectomy

The sequence of surgical maneuvers that should be used in each case will be dictated by the most proximal anatomic level reached by the TT inside the IVC. Thus, exposure of the right kidney and the infrahepatic IVC usually requires the Cattell-Braasch and extended Kocher maneuvers, while exposure of the left kidney necessitates the Mattox maneuver. In addition, gaining control of the TT within the IVC requires a complete circumferential dissection of this structure all along the entire length of the TT, since the presence of engorged tributaries entering through its posterior aspect can generate a massive intraoperative bleeding often difficult to manage. In addition to the ligation and division of the main renal artery of the involved kidney, it is necessary also to gain control of the IVC at three different levels: (i) at a level distal to the location of the most caudal end of the TT, (ii) at the ostium of the contralateral renal vein, and (iii) at a level cranial to the location of the most proximal end of the TT.

Generally, vascular control of the contralateral renal vein and caudal portion of the IVC does not represent a major problem. Cranial control of the IVC is rather more complex. Therefore, vascular control of the retrohepatic IVC below the MHVs can be obtained from an anterior approach using the exclusion maneuver described elsewhere by our group (RIVCA maneuver) [15]. Levels IIIa-IV will require complete exposure of the IVC and control of the hepatic hilum (Pringle maneuver). Gaining access to the retrohepatic and suprahepatic-infradiaphragmatic IVC requires the mobilization of the right hepatic lobe towards the midline (Langenbuch maneuver) or both hepatic lobes (i.e., complete liver detachment in a “Piggy-back” fashion). Furthermore, the circumferential control of the intrapericardial IVC and the caudal portion of the right atrium needs opening of the central tendon of the diaphragm, and frequently the ligation of both anterior diaphragmatic veins (commonly engorged in higher thrombi levels).

Once the caudal and contralateral venous control has been achieved, attention should be pointed to the proximal IVC. In this regard, one of the main objectives should be early reestablishing the natural venous shunt through the liver, aimed at maintaining cardiac preload. Sometimes, IVC clamping for cranial TT control is poorly tolerated by the patient. If the venous hepatic shunt can be reestablished in a short period of time, cardiac preload may be maintained in the interim by means of a rapid infuser. Otherwise, cardiopulmonary bypass initiation is strongly recommended.

For TT levels IIIb-IV, the milking maneuver can be used, trying to place a clamp below the level of the MHVs once the proximal end of the TT has been moved to this location. This maneuver should be performed under complete visual guidance provided by real-time transesophageal echocardiography (TEE), which confirms the complete TT mobilization downwards. In completely/almost completely occlusive TT, it is sometimes difficult to mobilize the cranial end to a level below the confluence of the MHVs due to the significant tumor burden present at this level. In these cases, opening the central tendon of the diaphragm is sometimes necessary to gain access to the intrapericardial IVC and the lower portion of the right atrium, which in so doing can even be mobilized to an intra-abdominal location in order to facilitate the proximal clamp placement. In these cases, it is often necessary to perform a two-stage cavotomy along with Pringle maneuver (i.e., to control the liver blood inflow) to remove the most cranial extent of the TT to early restore the natural venous shunt through the liver.

**Fig. 1:**
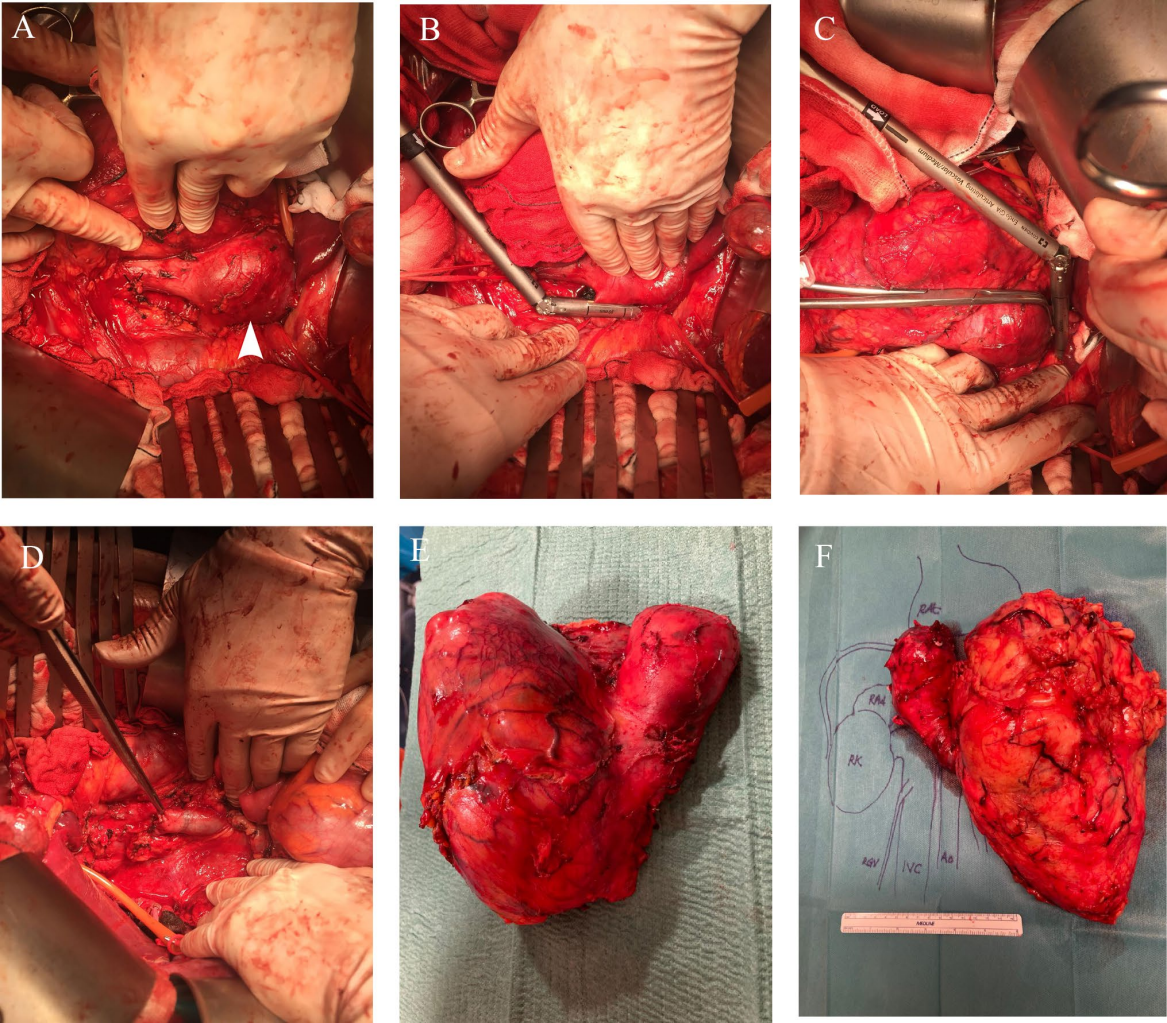
**A **Inferior vena cava exhibiting a completely occlusive tumor thrombus (white arrow). **B** Stapling the left renal vein. **C** Stapling the inferior vena cava cranial to upper tumor thrombus limit. **D** Inferior vena cava has been removed en bloc with the nephrectomy specimen, the remaining infrarenal vena cava is shown. **E** Right renal cell carcinoma removed en bloc with the inferior vena cava. **F** Left renal cell carcinoma removed en bloc with the inferior vena cava

Stapling of the IVC can be performed without difficulty using an endo-GIA at the infrarenal level. If the intention is not to replace the IVC, it is necessary to preserve as much as possible the existing collaterals that will facilitate the drainage of the lower body. Stapling the left renal vein is performed easily either, given the presence of abundant tributaries through which drainage will adequately occur. The case of the right kidney is rather more complex, since it lacks tributaries for venous drainage. If posterior tributaries close to the ostium of the right renal vein exist, these may constitute an optimal drainage alternative. The absence of these tributaries almost inevitably implies the need to reconstruct the drainage of the right kidney, either by maintaining the patient IVC (i.e., without performing circumferential cavectomy) once the TT has been already removed or by replacing the IVC with a ringed expanded polytetrafluoroethylene (ePTFE) prosthesis. Finally, proximal stapling should be performed preferably in an oblique fashion below the location of the MHVs, thus avoiding thrombosis and ensuring adequate venous drainage of the liver (Fig. 1).

## Initial Experience at Our Center

In the period of 2018–2023, a total of 13 nephrectomies associated with thrombectomy and circumferential cavectomy without caval replacement were performed at our center following the technical steps described above. The demographic, oncologic, and comorbidity characteristics of the patients prospectively included in this series are shown in Table 1.

**Table 1:** Main demographic, disease, surgical features, and complications of the patients included in the series

Case #number	#1	#2	#3	#4	#5	#6	#7	#8	#9	#10	#11	#12	#13
Demographics													
Age (years)	71	59	66	65	69	62	77	74	48	72	74	54	58
Gender	M	M	M	M	M	M	M	M	F	M	M	M	M
BMI (kg/m2)	26.2	31.3	27.8	24.7	27.1	31.5	28	24.2	22.7	29.1	24.5	23	24.5
Comorbidity													
Charlson comorbidity index	11	9	9	8	10	7	8	11	14	18	9	8	7
Karnofsky status (%)	70	90	80	80	90	90	90	80	90	80	70	70	90
ECOG-PS score	2	1	1	1	1	1	1	1	1	1	2	1	1
ASA score	III	II	IV	III	III	IV	III	IV	II	III	III	III	III
Disease features													
Symptoms	L + S	L + S	L + S	L	S	L	S	L + S	L	S	L + S	L + S	S
Preoperative PE	-	-	Y	-	-	-	-	Y	Y	-	-	Y	Y
Size (cm)	11.2	10.4	7.2	14	11	14	4	7	14	8	12.3	13	11
Laterality	R	R	R	R	R	R	R	R	R	R	L	R	L
Tumor thrombus level (UMCS)	IIIa	IIIa	IIIb	IIIa	IV	IV	IIIa	IIIa	IV	IIIa	IIId	IV	IIIc
Pathology	Cl	Cl	Cl	Cl	Cl	Cl	Cl	PC type II	Cl	Cl	Cl	Cl	Cl
Special features	RD	RD	SD	RD	-	SD	-	-	RD	-	RD + SD	-	-
ISUP score	IV	IV	IV	IV	III	IV	III	III	IV	III	IV	III	IV
Metastases	+	-	-	+	+	+	-	-	+	+	+	-	-
Surgery													
CPB	-	-	-	-	Y	Y	-	-	-	-	-	Y	-
Operative time (min)	260	180	240	190	350	260	230	200	280	190	210	300	260
Complications													
Major PO complications, 30 days (Clavien-Dindo)	-	-	-	-	IIIa	IIIa	-	-	-	-	-	IVb	-
Cause	-	-	-	-	(*)	(**)	-	-	-	-	-	(***)	-
Intraoperative PE	No	No	No	No	No	No	No	No	No	No	No	No	No
Hb preoperative (g/dL)	10.5	11.4	10.1	14.9	10	9.7	14.3	7.4	10.6	11.6	9.7	10.9	11.1
Hb postoperative (g/dL)	9.6	9.6	10.5	13	7.3	9.5	10.7	9.3	9.3	8.4	9.6	10.6	9.6
EBL (cc)	1000	1500	1500	500	8000	7500	700	1500	2000	2500	2500	25,000	1500
PRBC units intraop	0	0	2	0	8	13	0	2	2	0	1	45	3
PRBC units PO	2	2	1	0	8	2	0	4	2	1	4	5	0
Total transfusion (PRBC units)	2	2	3	0	16	15	0	6	4	1	5	50	3
LOS (days)	8	9	9	9	47	16	25	36	32	14	9	80	20

*M* male, *F* female, *L* local, *S* systemic, *R* right, *L* left, *BMI* body mass index, *Cl* clear cell carcinoma, *PE* pulmonary embolism, *PC* papillary carcinoma, *RD* rhabdoid differentiation, *SD* sarcomatoid differentiation, *CPB* cardiopulmonary bypass, *PRBC* packed red blood cells, *ISUP* International Society of Urological Pathology, *PO* postoperative, *EBL* estimated blood loss, *LOS* length of stay

(*)Hemoperitoneum requiring surgical revision (bleeding from the nephrectomy surgical bed), postoperative pneumonia

(**)Atrial fibrillation requiring temporary pacemaker placement

(***)Right ventricular dysfunction/cardiac arrest in the context of preoperative pulmonary embolism. Budd-Chiari syndrome. Severe after CPB initiation requiring politransfusion

The diagnosis of disease and IVC involvement was based on the level of TT observed on preoperative CT. In cases where the upper limit of the tumor thrombus was not clearly depicted, MRI and/or transthoracic echocardiography was performed to complete the initial cross-sectional evaluation. The cranial extent of the tumor was initially defined according to Neves and Zincke, while level III thrombi were subclassified according to the UM Classification System.

Surgical interruption of the IVC was performed either in the presence of a TT totally occluding the IVC not amenable to thrombectomy (i.e., impossible to separate the TT from the IVC vein wall), or in the presence of bulky bland thrombus below the TT impossible to be removed completely during the intervention. The presence of an adequate collateral venous network was always ascertained. Circumferential interruption of the IVC was performed en bloc with the diseased kidney by stapling with an endo-GIA at three different levels as explained before. None of the cases required IVC replacement. In all cases, collateral venous flow was preserved by minimizing the ligation of the venous tributaries encountered. Preoperative anticoagulation was prescribed in cases of associated bland thrombus, thromboembolic event prior to surgery, presence of atrial thrombus, or significant risk for PE. All patients received lower limb compression during surgery and until postoperative ambulation was verified.

Out of the 13 cases, 1 was woman (7.69%), and only 2 (15.4%) were left. The mean age was 66 years (range, 48–77). The mean body mass index was 26.2 kg/m2 (range, 22.7–31.5 kg/m2). The Charlson comorbidity index ranged between 7 and 18, ECOG score was predominantly 1 (84.6%), and Karnofsky status varied from 70 to 90%. All patients were considered fit for surgery and extensive counselling was obtained prior to surgery highlighting the potential complications expected during and after the intervention.

Presentation at debut was variable, but the majority of the patients complained of both local and systemic symptoms (53.8%). Preoperative lower limb edema or occluding symptoms were unfrequent (1/15 case, 7.1%). Preoperative PE was detected in 5/13 patients (38.4%) and resolved in 4 cases with anticoagulant therapy (60 IU enoxaparin/12 h) before the procedure. The mean tumor size in preoperative CT was 11 cm (range, 4–14). TT was classified as level IIIa-IV in 46.1% (6/13), 7.6% (1/13), 7.6% (1/13), 7.6% (1/13), and 30.7% (4/13), respectively. Concomitant metastases were visible in 53.8% (7/13) of the patients included in the series. Pathology analysis showed a conventional variant in most cases (92.3%). Special pathological features (i.e., sarcomatoid or rhabdoid differentiation) were present in 53.8% of the cases. International Society of Urological Pathology (ISUP) score was predominantly III-IV. Only one of the cases was classified as type II papillary carcinoma. Surgical margins were negative in all the patients included.

Stapled endo-GIA cavectomy between the MHVs and renal vein location (including the TT within) en bloc with the diseased kidney was completed in all cases, in a mean time of 240 min (range, 180–350 min). In cases of metastatic spreading to local lymph nodes, a standard lymph node dissection including all macroscopically involved lymph nodes was performed. Contralateral adrenalectomy was performed in two cases due to concomitant metastatic involvement. Median estimated blood loss (EBL) was 2500 cc (range, 500–25,000 cc). Transfusion was administered upon discretion of the treating team, but commonly with a lower threshold of hemoglobin ≤ 7 g/dL, and was required in 11/13 patients (84.6%) either during or after the procedure. The median total transfusion was two units of packed blood red cells (PBRC). Notably, blood requirement was higher in patients requiring cardiopulmonary bypass (CBP) for procedure completion despite the use of a cell saver device.

No deaths were seen in this series. Transient renal insufficiency (temporary decreased of glomerular filtration) was detected in approximately one half of the patients included (7/13 cases; 53.8%). Renal function stabilized soon after the procedure (i.e., during the first 3 months after the procedure) in all cases. Lower limb edema was seen only in two cases (15.3%) and resolved by the time of discharge.

Major postoperative complications were detected in 3/13 patients (23%). One of the patients with a level IV thrombus exhibit hemoperitoneum requiring surgical revision. No major vascular structure was seen involved, but conversely, extensive bleeding from the surgical bed was detected probably secondary to postoperative anticoagulation therapy required after CPB use. Surgical packing and further revision were enough to successfully control bleeding. Other patient with a level IV thrombus, also operated under CPB, developed a postoperative atrial fibrillation requiring temporary pacemaker implantation. The device was successfully removed at postoperative day 12. Finally, one of the patients with a level IV thrombus developed a Budd-Chiari syndrome before the procedure. The patient presented also a large PE at the right pulmonary artery that did not dissolve with enoxaparin preoperatively. The procedure was almost completed exclusively from the abdomen, but when Pringle maneuver was released, the right ventricle could not manage the increased preload from the liver circuit due partly to right pulmonary artery occlusion. CPB was initiated, and right pulmonary endarterectomy was performed. The patient finally developed a multifactorial severe coagulopathy requiring a total of 50 units of PRBC among other products. However, he is alive and fully recovered at the time of this review.

In summary, radical nephrectomy en bloc with circumferential IVC and TT removal is a safe procedure at centers with enough experience, given that major complications observed in our experience are unfrequent and manageable when they occur. From an oncological standpoint, every vestige of visible disease was removed during the intervention satisfactorily. IVC replacement in these cases is seldom necessary according to our experience, since no established renal insufficiency or long-lasting lower limb edema was seen in our series. Transfusion is common and clearly linked to CPB use, as so major complications are. However, sometimes complete disease removal cannot be achieved without CPB use. Although this intervention represents a surgical challenge not exempt of major complications, it can provide for some survival extension, severe reduction in tumor burden that would improve response to systemic therapy, and improved quality of life. 

## Data Availability

No datasets were generated or analysed during the current study.
